# *In vivo* imaging of tau deposition in Alzheimer’s disease using both [^18^F]-THK5317 and [^18^F]-S16: A pilot human study

**DOI:** 10.3389/fnagi.2022.994750

**Published:** 2022-08-26

**Authors:** Liping Fu, Jinming Zhang, Kaixiang Zhou, Xiaojun Zhang, Hengge Xie, Mingwei Zhu, Mengchao Cui, Ruimin Wang

**Affiliations:** ^1^Department of Nuclear Medicine, China-Japan Friendship Hospital, Beijing, China; ^2^Department of Nuclear Medicine, First Medical Center, Chinese PLA General Hospital, Beijing, China; ^3^Key Laboratory of Radiopharmaceuticals, Ministry of Education, Beijing Normal University, Beijing, China; ^4^Department of Neurology, The Second Medical Center, Chinese PLA General Hospital, Beijing, China

**Keywords:** Alzheimer’s disease, tau tangles, positron emission tomography, [^18^F], THK5317

## Abstract

**Objective:**

To evaluate the effectiveness of a new tracer (*S*)-1-(4-(6-(dimethylamino)quinoxalin-2-yl)phenoxy)-3-fluoropropan-2-ol ([^18^F]-S16), in distinguishing patients with AD from HCs.

**Methods:**

Paired [^18^F]-S16 and [^18^F]-THK5317 scans were acquired in five patients with AD, six HCs, one subject with a semantic variant of primary progressive aphasia (sv-PPA) and one subject with probable progressive supranuclear palsy (PSP). Dynamic PET scanning was performed over 90 min after injection of the tracers. Standardized uptake values (SUV) and cortical-to-cerebellum standardized uptake value ratios (SUVRs) were used for tau deposition semi-quantization. A voxel-based analysis was employed to assess the uptake difference between populations.

**Results:**

[^18^F]-S16 exhibited excellent blood-brain-barrier penetration. AD patients showed increased cortical [^18^F]-THK5317 and [^18^F]-S16 binding. Compared to HCs, AD patients showed significantly increased cortical [^18^F]-S16 uptake in the bilateral occipital cortex, posterior cingulated cortex/precuneus, and lateral frontal cortex. Notable [^18^F]-S16 uptake was observed in the basal ganglia and brainstem compared to the neocortex. A substantial [^18^F]-S16 signal was detected in the basal ganglia and midbrain in a patient with probable PSP and in the bilateral anterior temporal cortex in a sv-PPA patient.

**Conclusion:**

[^18^F]-S16 might be of help to detect tau protein *in vivo*.

## Introduction

Alzheimer’s disease (AD) is a progressive neurodegenerative disease showing increasing incidence in the older population. Neuropathologically, AD is characterized by accumulating deposition of senile plaques and neurofibrillary tangles (NFTs), which are misfolded aggregates of amyloid-β (Aβ) and hyperphosphorylated tau-protein (Braak and Braak, 1991). Apart from AD, abnormal aggregation of tau protein also is central to a family of protein-misfolding diseases called tauopathies, including primary age-related tauopathy, Pick’s disease, corticobasal syndrome (CBS), progressive supranuclear palsy (PSP), and chronic traumatic encephalopathy (Liu and Gong, 2008). Considering the stereotypical topography of NFTs in AD and regionally distinct NFTs in other tauopathies, imaging tau protein has aroused scientific and medical interest due to the possibilities for disease monitoring, evaluation of therapeutic outcomes, and differential diagnosis.

Currently, different classes of tau tracers such as flortaucipir, THK5351, PBB3, and MK6240 have been developed and utilized in previous clinical studies. It has been reported that the topographical distribution of tracer binding in AD follows the distribution of NFTs as seen in post-mortem analysis and shows a close relationship with the clinical severity and phenotype of dementia (Okamura et al., 2014; Marquié et al., 2017; Shimada et al., 2017; Xia et al., 2017; Villemagne et al., 2018; Betthauser et al., 2019). [^18^F]-THK5117 has shown a high affinity for and selective binding to tau pathology (Harada et al., 2015; Lemoine et al., 2015). Its *S*-form enantiomer (also known as [^18^F]-THK5317) shows favorable pharmacokinetics (Jonasson et al., 2016). Although the first-generation tau tracers are promising, the prominent off-target binding seen in the basal ganglia and midbrain may restrict their clinical application for non-AD tauopathies.

(*S*)-1-(4-(6-(dimethylamino)quinoxalin-2-yl)phenoxy)-3-fluoropropan-2-ol ([^18^F]-S16) is being developed as a diagnostic radiopharmaceutical to be used as a non-invasive assessment tool to aid the clinical evaluation of subjects with conditions associated with increased NFTs accumulation, such as AD. Preclinical study of the non-radioactive compound [^19^F]-S16 demonstrated excellent fluorescent properties and high selectivity for NFTs in brain sections from tau transgenic mice and AD patients, which indicated that it might be a promising positron emission tomography (PET) tracer for tau imaging (Zhou et al., 2021).

Paired studies in the same subjects with pharmacokinetic analysis could provide valuable information derived from the resulting meaningful comparisons. The aim of this exploratory study was to assess the brain-imaging properties and tolerability of [^18^F]-S16 by directly comparing global and regional uptake of [^18^F]-THK5317 and [^18^F]-S16 in the same elderly healthy controls (HCs), AD patients, and young volunteers.

## Materials and methods

### Eligibility and study design

This clinical human study of [^18^F]-S16 was performed at the Chinese PLA General Hospital. The Thirteen enrolled individuals included five patients diagnosed with AD and six age-matched HCs. Two patients with non-AD dementia also were recruited as an exploratory step, including one with a clinical diagnosis of a semantic variant of primary progressive aphasia (sv-PPA) and one with PSP. All patients were referred to the memory clinic in the Department of Geriatric Medicine, Chinese PLA General Hospital. Patients with AD had a “probable AD” diagnosis according to the National Institute of Neurological and Communicative Disorders and Stroke and the Alzheimer’s Disease and Related Disorders Association, with a Mini-Mental State Examination (MMSE) score between 10 and 24, inclusively (Folstein et al., 1975; Alzheimer’s Association [AA], 2004), and positive results obtained with an amyloid PET (Carbon 11-labeled Pittsburgh Compound B, [^11^C]-PIB) scan. HCs were enrolled with no evidence of cognitive impairment based on history and psychometric testing, without a family medical history of AD, and had an MMSE score of at least 28. Patients with AD could be taking a stable dose of the acetylcholinesterase inhibitor, memantine, or vitamin E. None of the AD patients accepted anti-Aβ treatment. The two patients with non-AD dementia were clinically diagnosed as follows. One patient was diagnosed with progressive language disorder that mainly presented as impairment of word comprehension and naming, failure of retrieval, and vague speech, which met the criteria for sv-PPA (Gorno-Tempini et al., 2011). The other patient was diagnosed with progressive postural instability and falling, vertical supranuclear ophthalmoparesis, dysdiadochokinesia, and executive deficits, which fulfilled the criteria for probable PSP (Litvan, 2001). The plasma samples obtained from HCs and patients were available for laboratory tests to exclude possible interfering conditions, such as folic acid deficiency and thyroid hypofunction. All procedures were approved by the appropriate Institutional Review Board, and all participants or an appropriate representative signed the informed consent forms after receiving a complete written and verbal description of the study.

### Positron emission tomography/MRI scans

All subjects received [^18^F]-THK5317 and [^18^F]-S16 scans in random order over the course of two weeks. PET/MRI scans were performed using a SIEMENS (Biograph mMR, Germany) PET/Magnetic Resonance Imaging (PET/MRI) system. This hybrid scanner enabled the acquisition of 127 axial planes over a 25.8 cm axial field of view, which allows the whole brain to be imaged in a one bed position.

Ninety-minute dynamic scans for [^18^F]-THK5317 and [^18^F]-S16 were successfully performed in one AD patient and six HCs. The protocol for the dynamic scans included an initial MRI sequence acquisition for MRI-based attenuation correction (AC), intravenous tracer injection and was followed immediately by a dynamic PET scan. First, an ultra-short echo time (UTE) sequence was acquired with (TE1/TE2 = 0.07/2.46 ms, TR = 11.94 ms, flip angle 10°, 192 slices, matrix size: 192 × 192 × 192, FOV = 300 mm × 300 mm, voxel size 1.6 mm × 1.6 mm × 1.6 mm, acquisition time = 1:40 min/bed position). Next, a dynamic PET emission scan in a three-dimensional acquisition mode was started simultaneously with a single intravenous bolus of [^18^F]-THK5317 (range 333–518 MBq) or [^18^F]-S16 (range 315–576 MBq). Dynamic brain PET images were reframed and reconstructed into 59 time-series frames (30 × 5, 10 × 30, 5 × 150, 14 × 300 s). For both tracers, 40–60 min of acquired dynamic data was then reconstructed into static images for standardized uptake value ratios (SUVR) comparison (Chiotis et al., 2016; Jonasson et al., 2016). The remaining subjects, including four AD patients, one sv-PPA patient, and one PSP patient, underwent [^18^F]-THK5317 and [^18^F]-S16 PET/MRI scans with a 20-min static run (40–60 min), 40 min after injection dose of 4.5 MBq/kg.

Both the static (20min duration) and dynamic PET data were reconstructed using an ordered subset expectation maximization algorithm with settings of iterate = 3, subset = 21, matrix = 336 × 336, and a Gaussian filter of 4mm in full-width half-maximum (FWHM), and then were converted into standardized uptake value (SUV) and SUVR images for further analysis.

Following the UTE sequence for PET emissions AC, structural MRI scanning was performed simultaneously with PET imaging using the sequence protocol as previously described (Fu et al., 2021).

### Radiosynthesis of positron emission tomography tracers

All patients underwent a 20-min [^11^C]-PIB PET/MRI static scan prior to the tau imaging, which was performed 40 min after injection (40–60 min) of 4.5 MBq/kg (McNamee et al., 2009). The [^11^C]-PIB, [^18^F]-THK5317 and [^18^F]-S16 were synthesized from their corresponding precursors as described previously (Zhang et al., 2008; Fu et al., 2021; Wang et al., 2021). The radiochemical purity of three tracers were more than 95% and their specific activities ranged from 90 to 123.5 GBq/μmol, which was corrected at the end of synthesis.

### Image and data analysis

#### [^18^F]-S16 and [^18^F]-THK5317-positron emission tomography image pre-processing

The high-resolution T_1_ weighted MRI brain images were first segmented for brain tissue using the new segment method in Statistical parametric mapping 8 (SPM8, Wellcome Trust Centre for Neuroimaging) and spatially normalized to a custom template, generated using all the T_1_ images and the DARTEL registration method in SPM8. Normalization of the whole uptake [^18^F]-S16 and [^18^F]-THK5317 frames for each subject underwent two steps: (1) the frames of both tracers, which were aligned to the middle frame, were first co-registered with the MRI scans using normalized mutual information; (2) then, the aligned images were spatially normalized to the custom template space based on the spatial normalization transformation parameters determined from the MRI. Static PET (40–60 min) images were firstly aligned onto the individual T1-weighted images (T1WI) and then spatially normalized to the custom template using SPM8 and the transfer matrix generated from the spatial normalization process of T1W.

#### Derivation of standardized uptake value ratios

The automated anatomic labeling (AAL) atlas was adopted for extracting cerebellar regions. Using DARTEL, the AAL atlas was normalized to the custom MRI T1WI template in MNI space. By applying the inverse deformation created by DARTEL, the AAL atlas was warped to the subject’s native anatomical space. Then atlas-based parcellation of PET images into ROIs was performed in subject space. The cerebellar regions were placed over cerebellar gray matter, and the cortical ROIs were created in the high-blood-flow regions of the frontal, medial temporal, lateral temporal, parietal, occipital, posterior cingulate cortex, and cortical regions. SUVRs were calculated using cerebellar gray matter as the primary reference region.

#### Voxel-base analysis

All voxels in the spatially normalized PET [^18^F]-THK5317 and [^18^F]-S16 images were divided by the mean [^18^F]-THK5317 and [^18^F]-S16 uptake of the cerebellar GM ROI in each subject to construct uptake ratio images. A two-sample *t*-test was employed voxel-by-voxel to assess the difference in tracer uptake between populations. For multiple comparisons, a false discovery rate was adopted to control the expected proportion of false-positive results at *P* < 0.05 and cluster size less than 50 voxels were abandoned. The cut-off value of SUVR for PIB-PET positivity was 1.4 (Tanaka et al., 2020).

### Statistical analysis

We compared the median age and education across groups using a Kruskal–Wallis test. Differences in MMSE scores between the HCs and AD groups were tested using a two-sided Wilcoxon rank-sum test. Pair-wise group differences for the [^18^F]-THK5317 and [^18^F]-S16 SUVRs were reported as differences in medians with 95% confidence intervals and tested using two-sided two-sample *t*-tests. SPSS (version 17) and SPM software were used to analyze the demographic, functional, and imaging data.

## Results

### Subjects

Thirteen subjects were injected with the two tracers, including five AD patients, six elderly HCs, one sv-PPA patient, and one PSP patient. Baseline characteristics are summarized in Table 1. As expected, the baseline average MMSE was significantly lower in the AD patients than the HCs (17.24 ± 6.05 vs. 28.89 ± 0.78; *T* = 36, *P* = 0.00). The AD and HC groups were similar with respect to age (*H* = 0.66, *P* = 0.42), education (*H* = 0.002, *P* = 0.96), and weight (*H* = 0.24, *P* = 0.62).

**TABLE 1:** Clinical and demographic characteristics of all subjects.

Demographic	AD patients	HCs	sv-PPA	PSP
*n*	5	6	1	1
Age (Y)	69.83 ± 8.35	65.67 ± 5.00	67	60
Sex (M/F)	1/4	4/2	F	M
Education (Y)	11.75 ± 4.20	9.67 ± 3.74	9	12
PIB-PET	Positive (5)	Negative (6)	Negative	Negative
MMSE*	17.24 ± 6.05	28.89 ± 0.78	21	22
Weight (Kg)	63.00 ± 13.71	64.17 ± 9.68	52	65
Injected dose (MBq)				
[F18]-THK5317	321.53 ± 61.05	355.21 ± 56.12	233.10	341.51
[F18]-S16	311.54 ± 67.34	319.31 ± 38.85	222.37	301.78

*MMSE scores for AD patients vs. HCs were significantly different based on a two-sided Wilcoxon rank-sum test, *P* < 0.05. Data are means ± SD or numbers of subjects.

### Imaging findings

The average [^18^F]-THK5317 and [^18^F]-S16 time-activity curves (TACs) in SUV units from 0 to 90 min after tracer administration for an AD patient and a HC subject are shown in Supplementary Figure 1.

**FIGURE 1:**
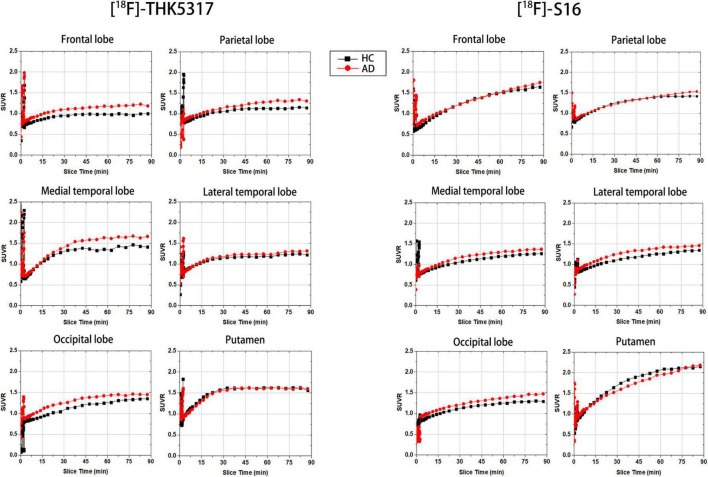
Time courses for regional SUVRs for two subjects in each diagnostic category.

Taken cerebellar GM as reference regions, the [^18^F]-S16 and [^18^F]-THK5317 SUVRs for an AD patient and a HC subject volunteer from the 0- to 90-min period are shown in Figure 1. The cortical retention of [^18^F]-S16 relative to the cerebellum was higher in the AD patient than in the HC subject, especially in the temporal and occipital regions. Despite the highest [^18^F]-S16 SUVR that was observed in the putamen in the AD patient, no evident differences were observed between AD and HC subjects. During the 90 min after administering [^18^F]-S16, the average cortical-to-cerebellar SUVRs demonstrated substantial continuous increases in the AD subject. The greatest difference in the SUVR between the AD and HC subjects was observed in the late stage of the total scan duration. For [^18^F]-THK5317, the average cortical-to-cerebellar SUVRs appeared to reach a steady plateau for both AD and HC subjects at 50-min after administration.

**FIGURE 2:**
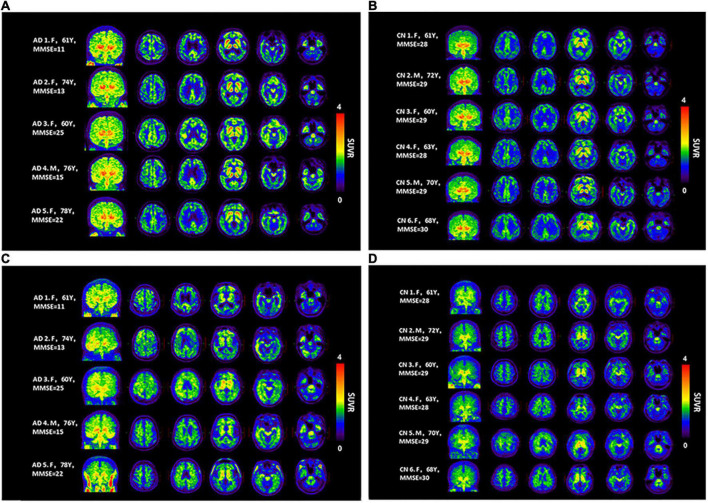
Maximum intensity projection (MIP) and axial [^18^F]-S16 **(A,B)** and [^18^F]-THK5317 **(C,D)** positron emission tomography images (40–60 min) for all Alzheimer’s disease patients (AD subjects 1–5) and healthy controls (HCs subjects 1–6). From the left to the right panels, the transverse PET images correspond to the levels at the centrum semiovale, PCC, basal ganglia, hippocampus, and basal temporal cortex, respectively.

Concerning the [^18^F]-S16 and [^18^F]-THK5317 visual assessment, the SUVR images from all subjects are shown in Figure 2. In the HCs, the cortical [^18^F]-S16 uptake was slightly greater than that in the white matter. In the AD subjects, the tracer accumulation was observed in cortical regions primarily involving the basal temporal cortex, frontal lobes, posterior cingulated cortex (PCC), and occipital lobe. In particular, the AD patient (subject #1) with the lowest MMSE score displayed the highest [^18^F]-S16 retention in the diffuse cortical regions. The [^18^F]-S16 uptake in the bilateral medial temporal cortex (MTC), especially in the amygdala, was identified in nearly all the subjects. [^18^F]-S16 retention was observed in the basal ganglia and brainstem of both HCs and AD subjects. No binding greater than the uptake in the reference region was observed in the choroid plexus and venous sinus. [^18^F]-THK5317 uptake in the cortical regions were identified in all AD patients. The radioactive retention in the basal ganglia, brainstem and eyeballs was observed in both AD and HCs subjects.

**FIGURE 3:**
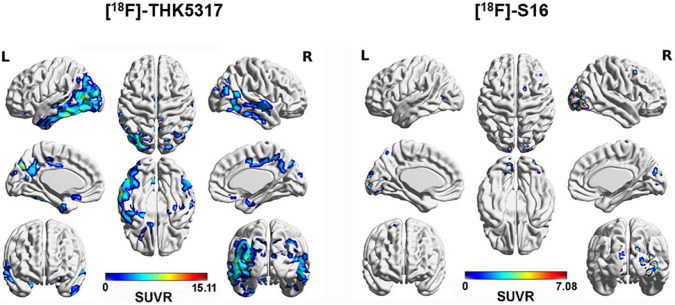
Surface projection maps for mean SUVR images comparing AD and HC subjects for [^18^F]-THK5317 and [^18^F]-S16. AD patients showed significantly increased cortical retention of [^18^F]-THK5317 in the bilateral temporal cortex, occipital cortex, PCC/precuneus, and prefrontal cortex. For [^18^F]-S16, increased radioactivity retention was observed in the bilateral occipital cortex, right parietal cortex, and right lateral frontal cortex.

In a voxel-wise analysis, compared to HCs, AD patients showed significantly increased cortical [^18^F]-S16 binding in the bilateral occipital cortex, PCC/precuneus, and lateral frontal cortex. [^18^F]-THK5317 displayed increased radioactive retention in the bilateral prefrontal and temporoparietal cortex (Table 2 and Figure 3).

**TABLE 2:** Voxel-based analysis between AD and HC groups in both [^18^F]-THK5317 and [^18^F]-S16.

Brain region	Voxel-level			Peak MNI coordinate		
		
	*T* value	Number of voxels	*P* value	X	Y	Z
[^18^F]-THK5317						
MTG, MOG, ITG, PCC, BA19, BA20, BA37, SOG	15.11	7620	0.001	−64	−42	−18
STG, BA21, MOG, ITG, BA37	6.54	2530	0.001	58	−6	0
Limbic lobe, Cingulate gyrus, Precuneus	7.41	796	0.001	−8	−28	46
PCC, Angular gyrus, SOG	6.14	431	0.001	24	−66	36
ITG, Temporal lobe	5.6	297	0.001	48	−14	−32
Limbic lobe, ParaHipp	9.82	180	0.001	34	−14	−28
[^18^F]-S16						
MOG, IOG, PCC/Precuneus, BA19	7.05	764	0.001	38	−86	14
Occipital lobe, Lingual gyrus	6.06	251	0.001	−14	−76	−4

Extent threshold: *k* = 100 voxel, voxel size: [2.0, 2.0, 2.0] mm.

MTG, middle temporal gyrus; STG, superior temporal gyrus; ITG, inferior temporal cortex; BA, Brodmann area; PCC, posterior cingulate cortex; SOG, superior occipital gyrus; MOG, middle occipital gyrus; IOG, inferior occipital gyrus; ParaHipp, parahippocampa gyrus.

**FIGURE 4:**
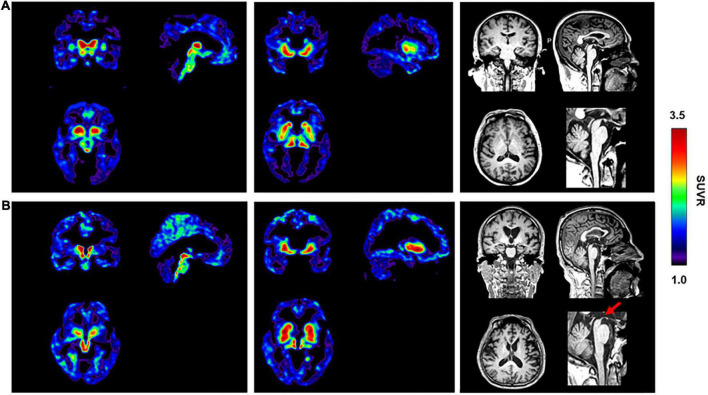
[^18^F]-S16 retention in an elderly healthy control [male, MMSE = 30, **(A)**] and a patient with progressive supranuclear palsy [male, MMSE = 18, PSP, **(B)**]. Transverse, sagittal and coronal sections of [^18^F]-S16 PET SUVR images were observed with considerable radioactive retention in the midbrain and basal ganglia. The typical “hummingbird sign” was detected on the sagittal T1 weighted image of the patient (red arrow).

The PSP and sv-PPA patients with non-AD dementia were amyloid-negative based on the absence of neocortical [^11^C]-PIB retention. Compared with the HC subjects (Figure 4A), the patient with the clinical diagnosis of probable PSP showed high radioactive retention of [^18^F]-S16 in the basal ganglia and midbrain (Figure 4B). The typical “hummingbird sign” was detected on the T1 weighted image of the patient, which indicated midbrain atrophy (Figure 4B). The patient with the clinical diagnosis of sv-PPA showed increased radioactive retention for [^18^F]-S16 and [^18^F]-THK5317, which was predominantly in the bilateral temporal pole, basal temporal cortex, and hippocampus (Supplementary Figure 2). Temporal atrophy was observed bilaterally on the T1 weighted image of the sv-PPA patient.

## Discussion

In this study, we first reported the results of a clinical trial using [^18^F]-S16, a new radioligand for tau tangle imaging, by directly comparing voxel-based cortical uptake of [^18^F]-S16 and [^18^F]-THK5317 in the same AD patients and HCs. [^18^F]-S16 was well-tolerated in all populations. No adverse events or clinically significant changes in the laboratory or vital parameters were observed. Healthy elderly controls demonstrated minimal [^18^F]-S16 retention, while AD patients demonstrated cortical distributions consistent with reported post-mortem data for tau tangles.

Using the cerebellum as the reference region (Marquié et al., 2015), the highest SUVRs for [^18^F]-THK5317 were observed in the MTC of the AD patient, followed by the parietal cortex and frontal cortex. Similar to [^18^F]-THK 5317, the greatest difference of [^18^F]-S16 SUVRs between AD and HC subjects was observed for the tempo-occipital cortex. The increased tracer uptake in the temporal and occipital cortices was consistent with the known distribution of NFTs found at autopsy in AD patients (Braak et al., 2006) and results from tau-PET imaging (Okamura et al., 2014; Marquié et al., 2017; Shimada et al., 2017).

It is noted that the [^18^F]-THK 5317 SUVRs for both AD and HC subjects reached a steady plateau at 50-min after administration, which was consistent with previous reports (Betthauser et al., 2017). In contrast, the SUVR for [^18^F]-S16 demonstrated a substantial, continuous increase during the 90 min of data acquisition for all subjects. Although a separation between the AD patient and the HC subject was observed, an asymptote with a plateau after injection, as seen with other [^18^F]-labeled tau imaging agents, was not observed (Xia et al., 2013; Betthauser et al., 2019). Even though a complete pharmacokinetic evaluation was not carried out, Wang and his colleagues performed a series of [^18^F]-S16 PET scans acquired at 10, 60, 120, and 240 min post-injection in AD and HC subjects and reported that the brain clearance rate was greater and the uptake ratio of WM/neocortex and WM/striatum increased further with time extension (Wang et al., 2021). Therefore, future research in which the total scan time is extended is needed to evaluate the pharmacokinetics and imaging features of [^18^F]-S16 more effectively and determine the optimal time window with the best discrimination between AD and HC populations.

As Figure 2 demonstrates, in AD patients, increased [^18^F]-S16 retention was primarily localized in the temporal-frontal-parietal cortex, which was consistent with results of NFT accumulation in post-mortem studies (Braak et al., 2006). Notably, the HC subjects showed relatively low [^18^F]-S16 accumulation in all cortical brain regions. Because of the limited number of AD patients in the current study, a correlation analysis between the MMSE score and [^18^F]-S16 SUVR was not performed. It was observed that patients with the lowest MMSE scores exhibited greater [^18^F]-S16 accumulation (AD subjects #1, #2, #4 vs. #3, #5), primarily involving the basal temporal cortex, orbitofrontal cortex, PCC, occipital cortex, and MTC. The spatial distribution observed in this study parallels neuropathologic data reporting the spread of tau from the entorhinal cortex through the inferior-lateral temporal and medial parietal areas to the neocortex (Braak et al., 2006).

In the voxel-based analysis for [^18^F]-THK5317, a significant difference in cortical uptake was observed between the groups, which corresponds with the known distribution of tau accumulation in post-mortem tissue and PET studies of [^18^F]-THK 5317 *in vivo* (Chiotis et al., 2016). AD patients also exhibited higher [^18^F]-S16 retention in the bilateral occipital cortex, and local parietal and frontal cortex, but the significant intensity and extent of the clusters were considerably less than [^18^F]-THK5317. Using ROI-based analysis and a 70–100 min post-injection static image acquisition, Wang et al., reported that the cortical [^18^F]-S16 SUVR of AD patients was significantly higher than that of HCs, and primarily involved the parietal, temporal, precuneus, posterior cingulate, and frontal lobes (Wang et al., 2021). We postulated that several factors contributed to the difference. First, although [^18^F]-S16 displayed high affinity and selectivity for tangles over Aβ as well as sufficient blood–brain barrier penetration and rapid brain washout in normal mice (Zhou et al., 2021), the 40–60 min time-window after [^18^F]-S16 administration that was used to measure the SUVR was not temporally stable in humans. Second, no MRI T1W images were used in the previous study to assist the confirmation of the reference region for SUVR calculation and the accuracy of the spatial normalization of PET images. Third, without partial-volume effect correction in the current study, the brain atrophy of AD might contribute to the underestimation of the [^18^F]-S16 PET signal and confound the hot-spot imaging analysis (Matsubara et al., 2016). The significant increased [^18^F]-S16 accumulation in the occipital cortex in AD patients might related to the late and less atrophy than the other cortical regions. Lastly, the limited number of recruited subjects decreased the statistic power.

As reflected from TACs and SUVR images, both [^18^F]-THK5317 and [^18^F]-S16 displayed the strongest uptake in the basal ganglia and brainstem in the AD and HC subjects. It is well known that the off-target binding in the basal ganglia, midbrain, choroid plexus, and eyeball could be observed with THK compounds, [^18^F]-AV1451, and [^11^C]-PBB3 images in *in vivo* studies, which likely reflects off-target binding to various entities, including monoamine oxidase A (MAO-A) and MAO-B, or pigmented or mineralized vascular structures (Lowe et al., 2016; Ng et al., 2017; Vermeiren et al., 2018). A previous study indicated that the [^18^F]-S16 shared the same THK binding site as NFTs (Zhou et al., 2021). However, following a series of successful optimizations and modifications, compared with [^18^F]-THK5351, the [^18^F]-S16 displayed numerous advantages, such as high binding affinity, selectivity, initial brain uptake, and low off-target binding (Zhou et al., 2021). Although [^18^F]-S16 exhibited poor MAO-B affinity (IC50 > 10 μM) in an animal study (Zhou et al., 2021) and lacked off-target binding in the eyeball in this human PET study, [^18^F]-S16 binding in the MAO-B-rich basal ganglia regions in subjects might be related to MAO-B binding. In addition, the off-target binding of [^18^F]-S16 in the basal ganglia region of subjects may be related to the *N*-dealkylation process. [^18^F]-S16 is an *N*, *N*-dimethylamino-substituted compound that undergoes *N*-demethylation in the basal ganglia which expresses higher levels of cytochrome P450 enzymes (Kawashima et al., 1996), which phenomenon was consisted with the Aβ tracers with *N*,*N*-dimethylamino group (Kung et al., 2010). The detailed contributions of possible specific and off-target [^18^F]-S16 signal sources in AD remain to be elucidated by correlative studies between PET and autopsy.

Although most tau PET studies have been performed in patients with AD, tau imaging is clinically valuable in other neurodegenerative conditions associated with tau pathology, such as PSP and CBD (Lebouvier et al., 2017). Although off-target binding of [^18^F]-S16 was observed in the basal ganglia in the HC subjects, relatively good discrimination abilities between the PSP and HC subjects were demonstrated due to the greater than expected [^18^F]-S16 accumulation in the regional pattern of tau pathology in the clinical diagnosis of the PSP patient, which was consistent with findings from [^18^F]-AV1451 and [^18^F]-THK5317 studies (Brendel et al., 2017; Cho et al., 2017; Schonhaut et al., 2017). sv-PPA, also called semantic dementia, is one of the clinical subtypes of frontotemporal lobe dementia (FTLD), which is characterized by progressive loss of semantic knowledge (Gorno-Tempini et al., 2011). In 69–83% of cases, the sv-PPA is associated with the underlying deposition of transactive response DNA-binding protein-43 (TDP-43) type C (Montembeault et al., 2018). For the remaining cases, AD pathology and FTD-tau also have been reported (Spinelli et al., 2017). In the current study, [^18^F]-S16 revealed strong binding in the anterior temporal lobe, which was consistent with the findings from other first-generation tau-PET tracers such as [^18^F]-AV1451 and [^18^F]-THK5351 (Josephs et al., 2018; Schaeverbeke et al., 2018). However, this was unexpected, as the major underlying pathology of sv-PPA is TDP-43. Schaeverbeke et al. suggested that [^18^F]-AV1451 and [^18^F]-THK5351 binding in sv-PPA is related to their affinity for MAO-B enzymes (Schaeverbeke et al., 2020). *In vitro* MAO-B inhibition binding measurements indicated that [^18^F]-S16 had poor MAO-B affinity, which suggested that the MAO-B off-target binding was not the main reason for tracer retention in the sv-PPA patient in this study. Given that the degree of frontotemporal atrophy in FTD is related to the degree of astrocytic apoptosis (Broe et al., 2004), it was expected that sv-PPA with FTLD-TDP would be characterized by extensive astrocytic apoptosis and astrogliosis (Schaeverbeke et al., 2020). The latter eventually becomes the overwhelming pathological feature as the disease progresses (Broe et al., 2004). Therefore, we postulated that [^18^F]-S16 demonstrated a strong signal in the sv-PPA patient *in vivo* due to the following two reasons. First, the female sv-PPA patient in the current study exhibited FTLD-tau pathology, and the target of [^18^F]-S16 was the tau protein. Second, considering that astrocytic apoptosis and astrogliosis existed during the disease process (Broe et al., 2004), the [^18^F]-S16 uptake might be associated with pathophysiological changes of sv-PPA. Therefore, the current cases of PSP and sv-PPA patients presented disease-specific [^18^F]-S16 uptake, which indicated that [^18^F]-S16 might be of great potential in the clinical utility of non-AD tauopathy.

One weakness of this pilot human study was that the number of subjects was not sufficient to provide definitive binding features for [^18^F]-S16 *in vivo* and to propose reliable differentiation for clinical purposes. A second potential weakness of this study was the relatively short data acquisition time, which did not allow evaluation of the complete kinetic information for [^18^F]-S16.

## Conclusion

Overall, [^18^F]-S16 was shown to be well-tolerated in this human study, with no serious adverse events observed. [^18^F]-S16 exhibited excellent BBB penetration in both AD patients and controls. Similar to the distribution of [^18^F]-THK5317, AD patients showed higher [^18^F]-S16 accumulation in the cortical target regions than HCs. One difference of [^18^F]-S16 was that it exhibited a substantial, continuous increase during the 90 min of data acquisition. Compared to HCs, AD patients showed significantly increased cortical [^18^F]-S16 uptake in the bilateral occipital cortex, PCC/precuneus, and LFC. Notable [^18^F]-S16 uptake was observed in the basal ganglia and brainstem compared to the neocortex, which indicated that off-target binding existed. However, the relatively good discrimination ability between the probable PSP patient and HC subject was revealed due to the increased [^18^F]-S16 accumulation that corresponded to the regional pattern of tau pathology.

## Data availability statement

The original contributions presented in this study are included in the article/Supplementary material, further inquiries can be directed to the corresponding authors.

## Ethics statement

The studies involving human participants were reviewed and approved by Chinese PLA General Hospital. The patients/participants provided their written informed consent to participate in this study.

## Author contributions

LF contributed to the research concept and design, data analysis and interpretation, drafting of the manuscript, and critical revision of the article and final approval of article. KZ and MC contributed to the data analysis and data interpretation. JZ and XZ contributed to the radiosynthesis and data analysis. HX and MZ contributed to the collection data and data interpretation. RW contributed to the research concept and design. All authors contributed to the article and approved the submitted version.
