# Supplementary figures and images for: In vivo imaging of tau deposition in Alzheimer’s disease using both [18F]-THK5317 and [18F]-S16: A pilot human study
Source: Front Aging Neurosci. 2022 Aug 26;14:994750. doi: 10.3389/fnagi.2022.994750 (PMC9459225; doi:10.3389/fnagi.2022.994750)

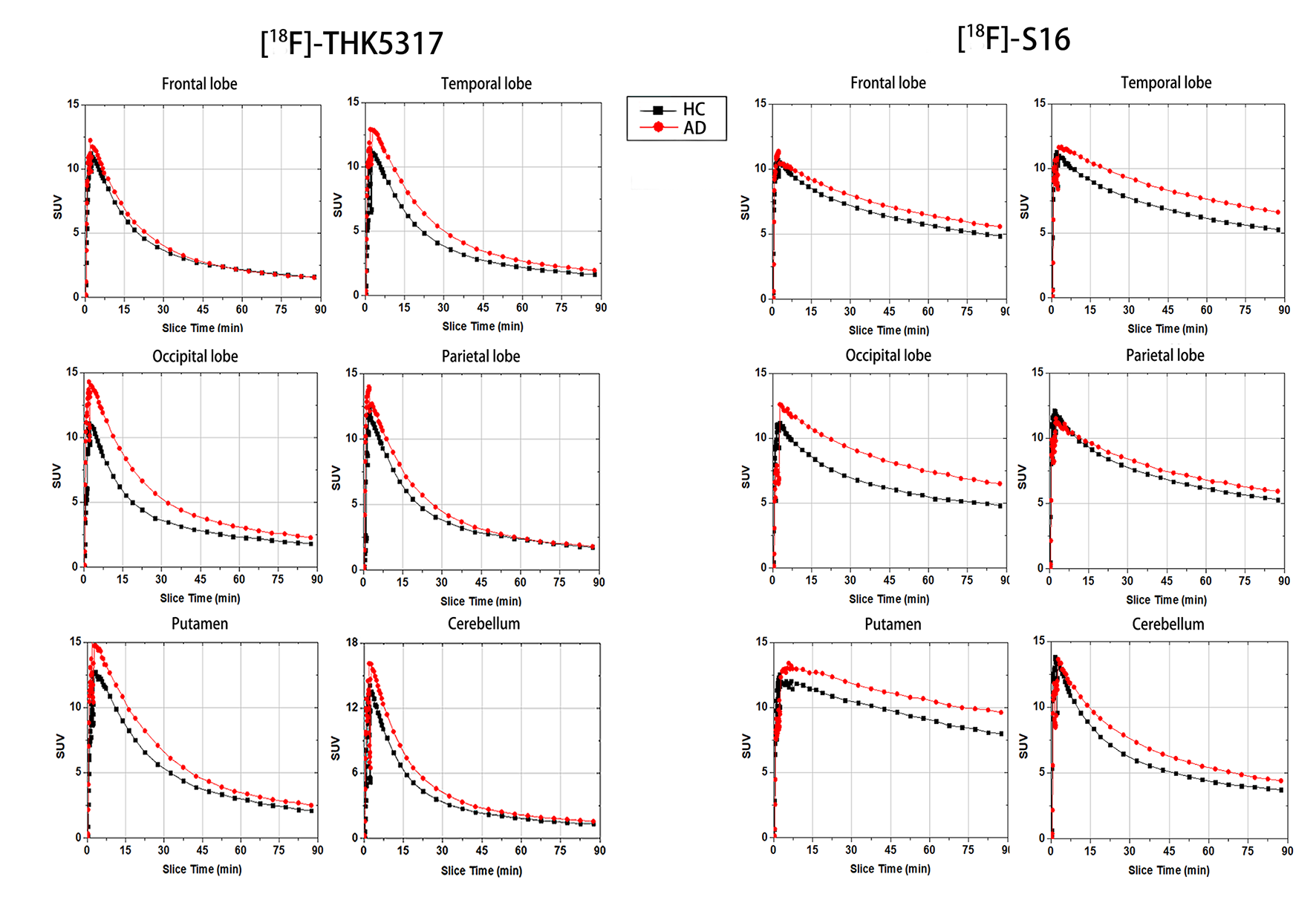

Supplement: Supplementary file 1 [file Image_1.TIF]

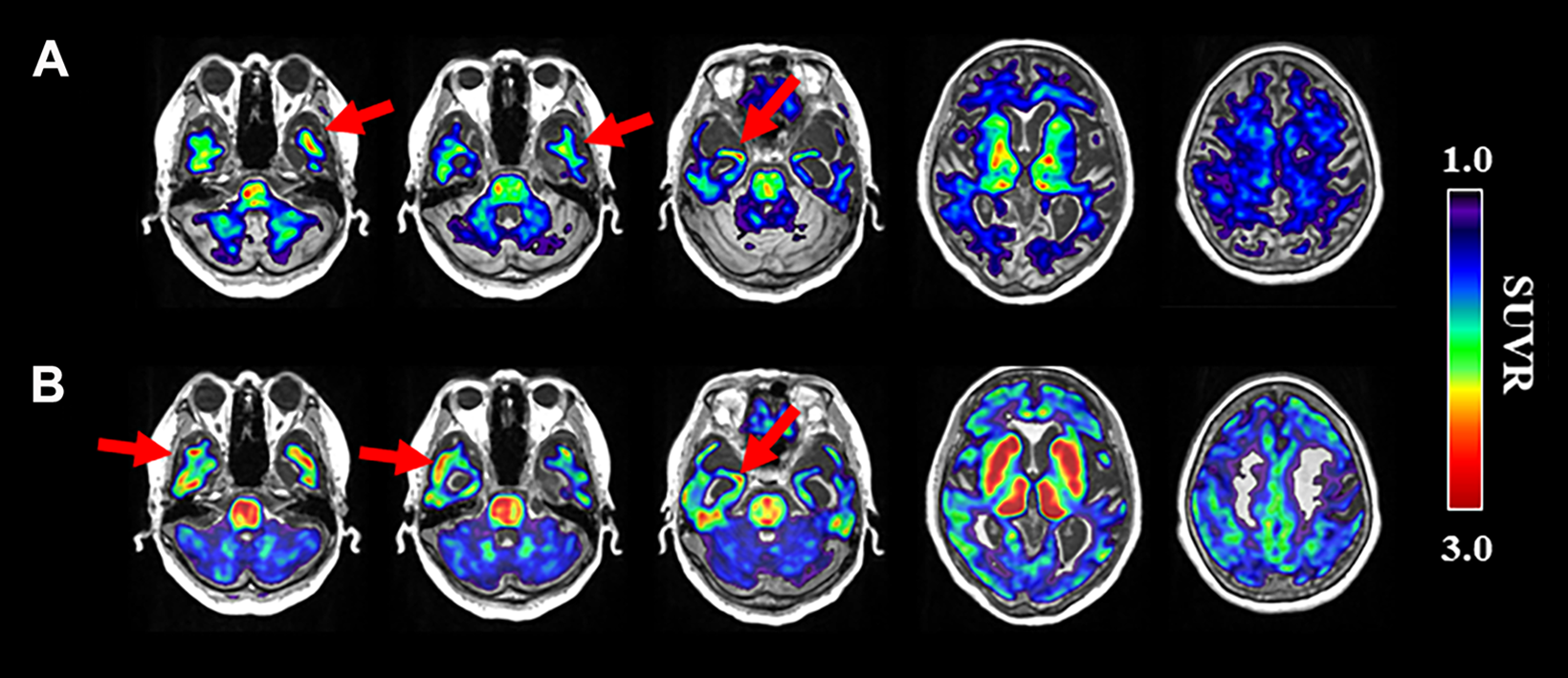

Supplement: Supplementary file 2 [file Image_2.TIF]
